# *Brucella melitensis* in France: Persistence in Wildlife and Probable Spillover from Alpine Ibex to Domestic Animals

**DOI:** 10.1371/journal.pone.0094168

**Published:** 2014-04-14

**Authors:** Virginie Mick, Gilles Le Carrou, Yannick Corde, Yvette Game, Maryne Jay, Bruno Garin-Bastuji

**Affiliations:** 1 Paris-Est University/Anses, EU/OIE/FAO & National Reference Laboratory for Brucellosis, Animal Health Laboratory, Maisons-Alfort, France; 2 Departmental Veterinary Laboratory of Savoie (LDAV 73), Chambery, France; East Carolina University School of Medicine, United States of America

## Abstract

Bovine brucellosis is a major zoonosis, mainly caused by *Brucella abortus*, more rarely by *Brucella melitensis.* France has been bovine brucellosis officially-free since 2005 with no cases reported in domestic/wild ruminants since 2003. In 2012, bovine and autochthonous human cases due to *B. melitensis* biovar 3 (Bmel3) occurred in the French Alps. Epidemiological investigations implemented in wild and domestic ruminants evidenced a high seroprevalence (>45%) in Alpine ibex (*Capra ibex*); no cases were disclosed in other domestic or wild ruminants, except for one isolated case in a chamois (*Rupicapra rupicapra*). These results raised the question of a possible persistence/emergence of *Brucella* in wildlife. The purpose of this study was to assess genetic relationships among the Bmel3 strains historically isolated in humans, domestic and wild ruminants in Southeastern France, over two decades, by the MLVA-panel2B assay, and to propose a possible explanation for the origin of the recent bovine and human infections. Indeed, this genotyping strategy proved to be efficient for this microepidemiological investigation using an interpretation cut-off established for a fine-scale setting. The isolates, from the 2012 domestic/human outbreak harbored an identical genotype, confirming a recent and direct contamination from cattle to human. Interestingly, they clustered not only with isolates from wildlife in 2012, but also with local historical domestic isolates, in particular with the 1999 last bovine case in the same massif. Altogether, our results suggest that the recent bovine outbreak could have originated from the Alpine ibex population. This is the first report of a *B. melitensis* spillover from wildlife to domestic ruminants and the sustainability of the infection in Alpine ibex. However, this wild population, reintroduced in the 1970s in an almost closed massif, might be considered as a semi-domestic free-ranging herd. Anthropogenic factors could therefore account with the high observed intra-species prevalence.

## Introduction

Bovine brucellosis is a major zoonosis, of worldwide public health and economic importance. The disease is mainly caused by *Brucella abortus* and, more rarely by *Brucella melitensis*, where cattle are kept in close association with infected sheep or goats. Infection is readily transmissible to humans and essentially acquired through direct contact with contaminated animal fluids or through the consumption of unpasteurized dairy products [1]. Infection can persist for long periods in both animals and humans.

France has been officially free of bovine brucellosis since 2005 and no brucellosis cases have been reported in domestic ruminants since 2003 [2]. As in most free countries, almost all human cases regularly diagnosed in France are imported from enzootic areas of other regions of the world. Rare autochthonous cases are either laboratory-acquired cases or due to a relapse months to years after therapy failure or to a reactivation of a latent initial infection.

**Figure 1:**
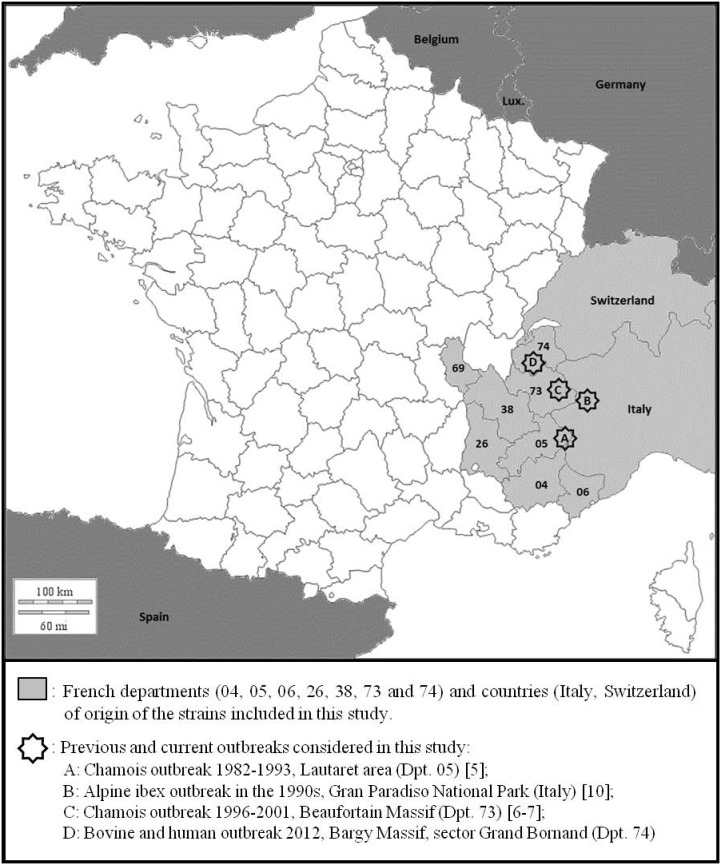
Survey area with current and previous *Brucella melitensis* bv 3 outbreaks in wild ungulates in the Alps.

In April 2012, a bovine brucellosis outbreak due to *B. melitensis* biovar 3 (Bmel3) occurred in a 21-head dairy herd producing raw milk cheese in the French Alps (Bargy Massif, Haute-Savoie (74), France) (Fig. 1), and was suspected as the source of an unexplained autochthonous human case previously diagnosed in January 2012 [3]. In-depth epidemiological investigations carried out in the area did not identify any potential source in domestic ruminants epidemiologically related to the outbreak. Moreover, according to the EU and French regulations, the whole domestic ruminant population is maintained under all-herd annual surveillance, particularly in regions where summertime transhumance is practiced; all imported animals are traced and controlled and must come from officially free herds, usually from officially free EU member states. In addition, France is a ruminant exporting country that imports very few numbers of animals per year. In Alpine area of concern, the production is very specialized and based on the exclusive use of local breeds without any introduction from other areas. All these elements strongly suggested that the source of this re-emergence was not due neither to autochthonous nor to imported domestic animals. This unexpected situation raised the question of a possible *Brucella* persistence and/or emergence in wildlife. An intensive investigation was therefore immediately launched (June-December 2012) in the extended neighborhood of the contaminated holding (a high-altitude mountain area) that provided new outstanding insights into Bmel3 infection in wildlife. The seroprevalence nearly reached 45% in Alpine ibex (*Capra ibex*) and almost 3% in Chamois (*Rupicapra rupicapra*) [4]. Additional investigations implemented in the neighboring massifs did not identify any other brucellosis case in wild ruminants (J. Hars, personal communication).

In the past, distinct isolated cases or outbreaks of Bmel3 infection had been reported in chamois in the French Alps between 1982 and 2001 (Lautaret, Hautes-Alpes (05), 6 cases [1982–1993]; Beaufortain, Savoie (73), 14 cases [1996–2001]) (Fig. 1) [5]–[7]. All these cases, as well as others reported in Spain or in Italy [8]–[10], could be related to pre-existing outbreaks in domestic ruminants in the area; further epidemiological investigations suggested a transmission from domestic animals to wildlife, due to interspecies cohabitation in the Alpine pastures. In addition, *Brucella* infection apparently remained localized, without extension to the nearby massifs, and infection seemed to disappear spontaneously, in no more than one generation timespan, after eradication of the domestic sources [8].

Wild ungulates, especially the chamois, have been therefore considered *(i*) as an epidemiological sentinel for the *B. melitensis* domestic infections and (*ii*) as an epidemiological dead-end, unable to sustain the infection, rather than a true reservoir [8]. Recently, *B. abortus* infection has been reported as self-sustained in elk and bison populations in the Greater Yellowstone area (USA) and spill-back has been demonstrated especially from elk to cattle [11]–[12].

In the absence of a significant infectious source in domestic ruminants, the very high prevalence of brucellosis in ibex suggested a possible self-sustained infection for year(s) within the wild population, including the chamois.

MLVA-16 (Multiple-Locus Variable-number tandem-repeat Analysis) assay [13] is at present the optimal strategy for *Brucella* molecular typing. The purpose of this study was to assess the genetic relationships among the Bmel3 strains possibly epidemiologically related to the recent outbreak, isolated from human, domestic animals and wildlife in the region and over a two-decade period and to propose a possible explanation of the origin of the recent bovine and human infections by the MLVA-panel2B assay.

## Materials and Methods

### Bmel3 Strains

Details on the bacterial strains used for this study are provided in Supporting information (Table S1). All animal strains were isolated as previously described [14], and biotyping was based on CO_2_ requirement, H_2_S production, urea hydrolysis, oxidase test, agglutination with monospecific sera (anti-A, anti-M, and anti-R), dye sensitivity (basic fuchsin and thionin), and phage typing (Tb, Wb, Iz, R/C) [14].

The Bmel3 strains isolated in April 2012 from 2 dairy cows (HS02, HS03) of the recent bovine outbreak in the French Alps (Bargy Massif, Dpt. 74, France) (Fig. 1) and from the human patient (HS01) diagnosed in January 2012 were included in this study [3]. Seven strains isolated during the investigation implemented in the area in wildlife (end of 2012) [4] from one chamois (HS04) and from four Alpine ibex (HS05-HS10) were included. Four of these wild animals showed chronic indurated lesions of arthritis on the foreleg joints from which strains (HS04, HS05, HS08 and HS09) were isolated. In addition, for two of them, ORD83 and ORD84, strains were also isolated from urine (respectively HS06 and HS07). A strain was isolated only from a vaginal swab, from the remaining ibex (HS10).

Sixty-five Bmel3 isolates from ANSES collection, collected from wild and domestic animals –dogs, cattle, sheep, goats, chamois, Alpine ibex– and from humans, from 1989 to 2007, in the outbreak area and the adjacent departments to the recent outbreak –Alpes-de-Haute-Provence (04), Hautes-Alpes (05), Drôme (26), Isère (38), Rhône (69), Savoie (73) and Haute-Savoie (74)– where summer transhumance is historically practiced, were included in this study (Fig. 1). Since the Alpine ibex is able to move on very long distances, an Italian Alpine ibex strain, isolated in 2005 from the Gran Paradiso National Park outbreak (Italy) (Fig. 1), as well as a Swiss human strain, isolated in 2002, were also included in this study. Two additional reference strains *B. melitensis* biovar (bv) 1 strain 16 M (ATCC 23456) and Bmel3 strain Ether (ATCC 23458) were included as controls.

### Molecular Typing and Data Analysis

Genomic DNA was extracted from *Brucella* cultures using the High Pure PCR Template Preparation Kit (Roche Diagnostics, France), according to the manufacturer’s instructions.

Twenty-five isolates were first characterized by MLVA-16, as previously described [13], [15]. MLVA-16 targets 16 genetic markers, organized in panels, to reflect distinct evolution rates. Panel 1 includes 8 minisatellites (bruce06, bruce08, bruce11, bruce12, bruce42, bruce43, bruce45, bruce55), more stable than panel 2 [8 microsatellites: 3 for panel 2A (bruce18, bruce19, bruce21) and 5 for panel 2B (bruce04, bruce07, bruce09, bruce16, bruce30)]. Subsequently, a fine scale genotyping analysis adapted to this particular epidemiological framework was employed. Thus, the isolates (n = 77) of this study were all characterized by the MLVA-panel2B assay (5 genetic markers) [15]. Fragment sizes converted to tandem repeat unit (U) numbers were imported into BioNumerics v6.6 (Applied Maths NV) as a character data set. The cluster analysis was performed using the UPGMA algorithm (Unweighted Pair Group Method Algorithm) with Euclidean distance matrices.

All loci do not evolve at the same evolution rate. Genetic diversity (Hunter-Gaston diversity index [HGDI]) was therefore calculated (http://www.hpa-bioinformatics.org.uk/cgi-bin/DICI/DICI.pl). Values of the HGDI can range from 0.0 (no diversity) to 1.0 (complete diversity). A Minimum Spanning Tree (MST) was constructed with a categorical coefficient (with 1/HGDI weight) to determine the evolution minimum path from one strain to all others on the network.

The obtained MLVA patterns were compared into the database *Brucella*2012, hosted by University Paris Sud (Orsay, France): http://mlva.u-psud.fr/mlvav4/genotyping/. Panel 1 data result in the MLVA-8 genotype, while panel 1 data together with the panel 2A pattern determine the MLVA-11 genotype.

## Results

### Genetic Diversity of MLVA-16 Molecular Markers for Bmel3

Evolution speed of MLVA-16 within the Bmel3 studied population is reflected by the HGDI, that can range from 0.0 (no diversity) to 1.0 (complete diversity) (Table 1). Higher values mean a higher tandem repeat polymorphism among the compared MLVA patterns, *i.e*. a lower genetic stability of the marker.

**Table 1:** HGDI values obtained in this study compared to global HGDI values obtained from published and personal results.

	Locus	HGDI (this study)				Global HGDI*			
		Diversity Index (*n*)	CI	K	max(pi)	Diversity Index (*n*)	CI	K	max(pi)
**Panel 2B**	Bruce04	0.791 (*77*)	0.753–0.830	7	0.325	0.765 (*507*)	0.745–0.785	10	0.377
	Bruce07	0.679 (*77*)	0.610–0.748	5	0.481	0.676 (*507*)	0.641–0.710	10	0.518
	Bruce09	0.858 (*77*)	0.816–0.901	13	0.286	0.637 (*507*)	0.592–0.683	15	0.588
	bruce16	**0.865** (*77*)	0.829–0.901	10	0.273	**0.838** (*507*)	0.828–0.849	11	0.245
	bruce30	0.126 (*77*)	0.025–0.227	5	0.935	0.693 (*507*)	0.664–0.721	7	0.480
**Panel 2A**	bruce18	0.034 (*25*)	0.000–0.098	2	0.983	0.582 (*432*)	0.541–0.624	7	0.595
	bruce19	0.000 (*25*)	0.000–0.114	1	1.000	0.577 (*432*)	0.540–0.615	7	0.583
	bruce21	0.000 (*25*)	0.000–0.114	1	1.000	0.009 (*432*)	0.000–0.022	2	0.995
**Panel 1**	Bruce06	0.000 (*25*)	0.000–0.114	1	1.000	0.466 (*432*)	0.442–0.490	2	0.632
	Bruce08	0.000 (*25*)	0.000–0.114	1	1.000	0.085 (*432*)	0.049–0.121	4	0.956
	bruce11	0.000 (*25*)	0.000–0.114	1	1.000	0.000 (*432*)	0.000–0.017	1	1.000
	bruce12	0.000 (*25*)	0.000–0.114	1	1.000	0.093 (*432*)	0.056–0.131	3	0.951
	bruce42	0.000 (*25*)	0.000–0.114	1	1.000	0.663 (*432*)	0.654–0.672	4	0.370
	bruce43	0.000 (*25*)	0.000–0.114	1	1.000	0.504 (*432*)	0.479–0.529	4	0.595
	bruce45	0.000 (*25*)	0.000–0.114	1	1.000	0.000 (*432*)	0.000–0.017	1	1.000
	bruce55	0.000 (*25*)	0.000–0.114	1	1.000	0.466 (*432*)	0.442–0.490	2	0.632

HGDI: Hunter Gaston Diversity Index, which determines the variation of the number of repeats at each locus, and ranges from 0.0 (no diversity) to 1.0 (complete diversity); CI: Confidence Interval; K: Number of different repeats present at this locus in this sample set; max(pi): Proportion of strains having the most frequent repeat number at a given locus (range 0.0 to 1.0); n: strains number according to the considered locus; in bold: locus more variable for panel 2B.

*Global HGDI calculated from published [13], [15]–[20], [22]–[23] and personal results.

Polymorphism of the MLVA-11 (panels 1 and 2A) pattern in the setting of our local outbreak investigation was null. Indeed, HGDI values of panels 1 and 2A, calculated from 25 strains, were closed to zero. HGDI values for panel 2B, calculated from 77 strains, displayed a higher discriminatory power. The most variable loci (HGDI values given in brackets) were: bruce16 (0.865), bruce09 (0.858), bruce04 (0.791), bruce07 (0.679), whereas the bruce30 (0.126) was very little informative.

We extended the HGDI calculation including MLVA-16 data only for Bmel3 strains from the literature [13], [15]–[20]; [22]–[23] and from our strain collection (n = 432–507 according to the considered locus) (MLVA data not shown) (Table 1). As regards the global data, HGDI values ranged from 0.000 to 0.663 for panel 1 (most variable: bruce42) and from 0.009 to 0.582 for panel 2A (most variable: bruce18) (Table 1). For panel 2B, HGDI values ranged from 0.637 to 0.838 with the highest variability for bruce16, bruce04 and bruce30 (respectively 0.838, 0.765, 0.693).

### Genotypes and Clusters

The complete MLVA-16 (16 loci) was performed on nearly one third of the isolates of the study, *i.e.* 25 field strains. The unique MLVA-11 pattern (panel 1 and panel 2A) obtained (panel 1 with for bruce06, bruce08, bruce11, bruce12, bruce42, bruce43, bruce45 and bruce55: 3 U, 5 U, 3 U, 13 U, 1 U, 1 U, 3 U and 3 U respectively and panel 2A with for bruce18, bruce19 and bruce21: 7 U, 42 U and 8 U respectively) was compared in the database *Brucella*2012. It belonged to the West Mediterranean group (genotype 96), with the MLVA-8 (only panel 1) genotype 51.

**Figure 2:**
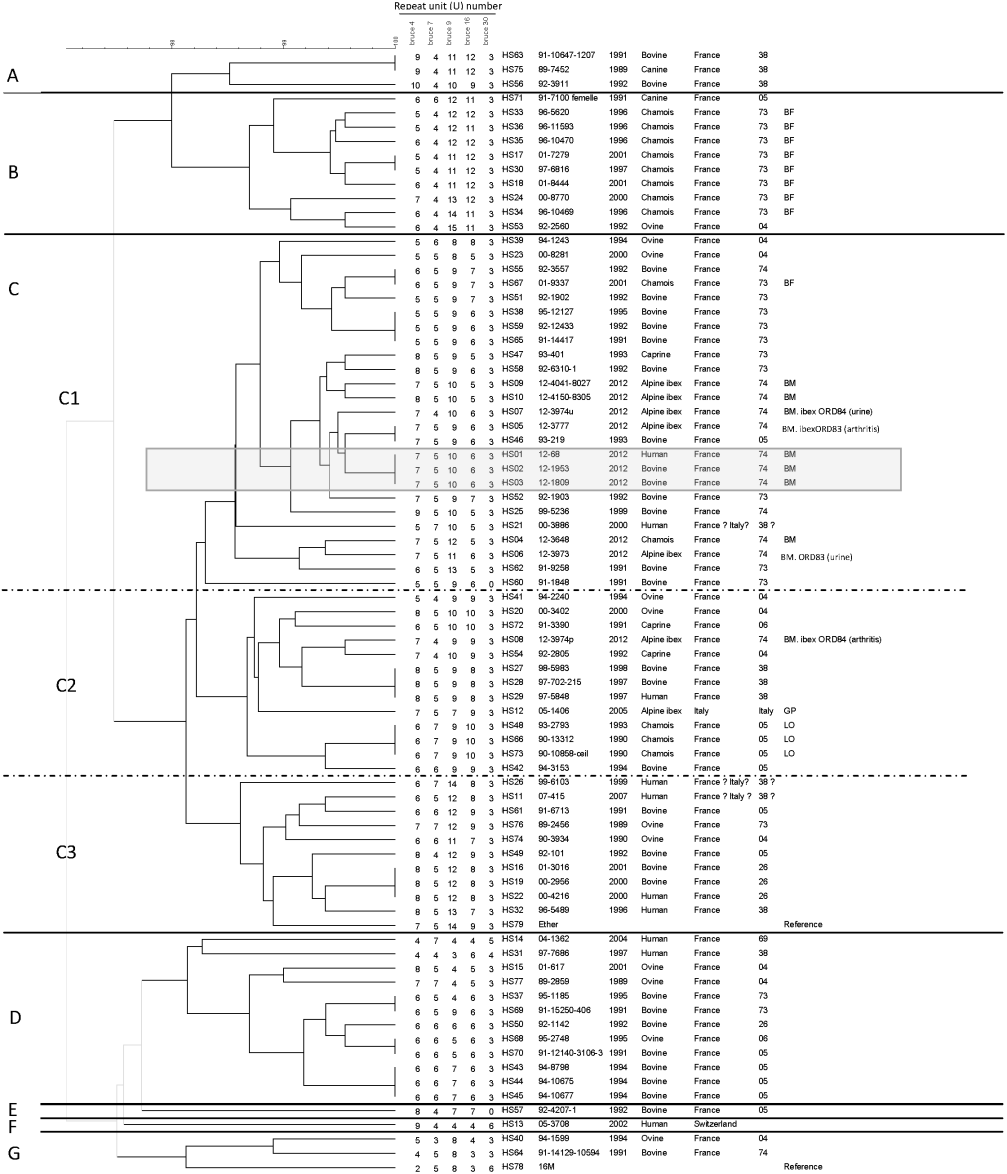
Dendrogram of clustered MLVA-panel2B genotypes (Euclidian distance, UPGMA algorithm). Clusters are coded as A-G, and sub-clusters as C1-C3. Isolates from the recent domestic and human outbreak are framed in a grey square. BM: Bargy Massif (*i.e.* the isolates from wildlife investigation campaign of 2012); BF: Beaufortain outbreak (1996–2001); LO: Lautaret outbreak (1982–1993); GP: Gran Paradiso outbreak, Italy (1990s). (Table S1).

MLVA-panel2B assay allowed clustering the 77 investigated strains (excluding the reference strains) into 61 genotypes (Fig. 2). With a similarity cut-off of 98%, the strains were distributed into 7 main clusters (Fig. 2). The major cluster C regrouped 62% of the isolates (49 strains, including the reference Bmel3 strain Ether), whereas the clusters A, B, D and G contained respectively 3.8% of the isolates (3 strains), 12.7% (10 strains), 15.2% (12 strains), and 3.8% (3 strains, including *B. melitensis* bv 1 strain16 M), and the singleton clusters E and F contained only one strain (1.25%).

The major cluster C was subdivided into 3 sub-clusters C1 (25 strains), C2 (13 strains) and C3 (11 strains). The isolates from the domestic and human outbreak 2012 (sub-cluster C1) showed a strictly identical genotype. These strains clustered with almost all the isolates from wildlife (ibex and chamois) in 2012, ranging from a single locus variant (SLV) (with only 1 U difference) (HS05, HS07, HS09) to a double locus variant (DLV) (with 1–2 U difference per locus) (HS10, HS04, HS06). In addition, strains of the 2012 domestic outbreak clustered also with domestic ruminants strains isolated in 1991–2001 in Dpt. 04, 05, 73 and 74, and in particular with a bovine strain (HS25) from the last outbreak reported on the same massif in 1999. Moreover, a strain isolated from ibex arthritis in 2012 (HS05) was genotypically identical with a bovine strain isolated from Dpt. 05 in 1993 (HS46).

In addition, with the exception of one chamois isolate (HS67, genotypically identical to a bovine strain from Dpt. 74: HS55; sub-cluster C1), the chamois strains isolated from the Beaufortain Massif (Dpt. 73) between 1996 and 2001 established a different cluster (cluster B). They were grouped with a dog and an ovine strains, respectively isolated from Dpt. 05 and Dpt. 04 (HS71 and HS53) (cluster B).

As regards the Lautaret outbreak (Dpt. 05), the chamois strains (HS73, HS66 and HS48) were grouped into the sub-cluster C2. Interestingly, this sub-cluster contained a recent ibex strain isolated from a caseous lesion (HS08), which showed a single variation (1 U) on bruce09 locus compared to a caprine strain from Dpt. 04 (HS54).

An identical genotype (sub-cluster C2) could be observed for two bovine strains isolated in 1997–1998 in Dpt. 38 and a human strain isolated the same year in the same region from an abattoir worker (HS27–HS29), suggesting an occupational disease. Moreover, no host specificity was noticed, as shown by the identical genotype for bovine and human strains (HS19, HS16 and HS22) (sub-cluster C3).

### Minimum Spanning Tree (MST)

**Figure 3:**
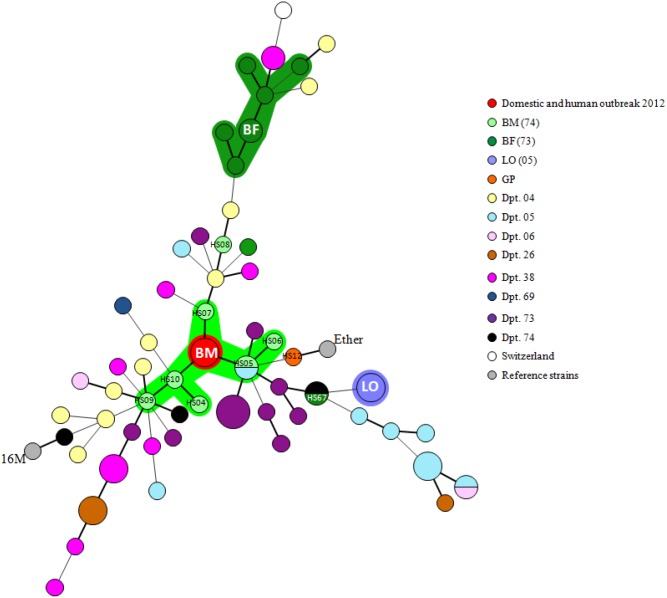
MST of clustered MLVA-panel2B genotypes. The MST was constructed with a categorical coefficient (with 1/HGDI weight for each locus). Size of circles reflects the number of isolates with a particular MLVA genotype. Width of the line reflects the genetic distance between the genotypes (heavy short lines connect single locus variants SLV, thin longer lines connect double locus variants DLV). Red circle, called BM for Bargy Massif, represents isolates from the recent domestic and human outbreak 2012. The strain codes are specified for recent cases (HS04-HS10) and other cited strains in results/discussion (*e.g*. HS67). Each department/country is assigned a different color, as well as the outbreak areas. BM: Bargy Massif (*i.e.* the isolates from wildlife investigation campaign of 2012); BF: Beaufortain outbreak (1996–2001); LO: Lautaret outbreak (1982–1993); GP: Gran Paradiso outbreak (1990s). (Table S1).

Phylogenetic relationships of Bmel3 isolates were revealed by a MLVA MST (Fig. 3). Results were consistent with the existence of various geographical sublineages. Interestingly, the recent domestic and human outbreak was in the centre of the network, directly connected with the Alpine ibex strains isolated during the 2012 investigation.

## Discussion

A recent (2012) bovine and human outbreak of Bmel3 in the French Alps (Bargy Massif) raised the question of a possible *Brucella* persistence and/or emergence in wildlife. The purpose of this study was to evaluate the epidemiological relationships between Bmel3 isolates collected from human, domestic and wild animals, over a two-decade period (1989–2012), in the direct surrounding areas of the 2012 outbreak and to investigate the origin of this unexpected event. A total of 77 Bmel3 field isolates have been investigated by the MLVA-panel2B assay. All strains of this study harbored the Western Mediterranean genotype 51 (MLVA-8) [13].

### Adaptation of MLVA-16 Methodology to a Local Epidemiological Setting

The MLVA-16 assay is adequate for macro-epidemiology of *Brucella* strains [13], [16], [21]–[23]. Nevertheless, within a restricted geographic area, panel 1–which allows the species-identification– as well as panel 2A present an uninformative polymorphism, as suggested by the HGDI values obtained in this study (close to zero), in agreement to the previously published investigations [21], [24]. The restricted genotyping scheme (only panel 2B, which contains the most variable loci) allows discriminating distinct outbreaks, clustering the strains known as epidemiologically-related and identifying a common infectious source. Our preliminary MLVA-16 studies on 25 field isolates of this study ensured the relevance of adopted markers. Diversity of the obtained genotyping patterns confirms that the MLVA-panel2B strategy is an efficient microepidemiological tool to discriminate strains isolated in a delimited geographical area, but it also highlights certain loci instability.

In several bacterial pathogens such as *Salmonella enterica* serovar Typhimurium, *Escherichia coli* O157:H7 or *Yersinia pestis*) [25]–[27], it has been reported that clinical, animal and environmental isolates, originated from the same epidemic outbreak, from the same patient (*i.e.* after antibiotic treatment), as well as after *in vitro* and *in vivo* stability assays, could harbor some small variations (SLV, DLV, etc.) in their MLVA patterns due to random genetic events, including DNA insertions, deletions, and point mutations. Their mutation rates were locus-specific [28]. These genetic events may impact the interpretation of MLVA data for epidemiological investigations and oblige to define an empirical cut-off for a correct interpretation. Noller and collaborators suggested that *E. coli* O157:H7 isolates that differ at no more than one locus with two-repeat units difference on 7 loci were highly related and belong to the same outbreak [29]. Also, a cut-off of a 2-U-difference at one or fewer loci was defined for *S. enterica* serovar Typhimurium isolates, with a VNTRs analysis on 5 loci [25]. Some investigations about *B. melitensis* Rev.1 vaccine strains estimated the genetic instability by MLVA-15 assay and showed variations in the number of repeats only at one or two loci (1–2 U) of the hypervariable panel 2B (bruce07, bruce09 and/or bruce16), and rarely in the bruce18 of panel 2A (1 U) [30]. In addition, a double amplification corresponding to two distinct alleles among the most genetically variable loci can be occasionally reported within a same isolate (data not shown), reflecting a mutation event, generated in the adaptation response of the microbial population in the host. In this study, we have chosen to report the most intense fragment as the allele designation.

Data from this study could lead to propose an interpretation cut-off in this particular local epidemiological setting: an isolate could be considered to be closely related to the outbreak strain or derived from the same source if a maximal total 3 U-difference is observed, especially in the most variable loci (higher HGDI). In this study, the most variable loci were: bruce16, bruce09 and bruce04. Nevertheless, additional investigations are required to develop *Brucella* specific guidelines for MLVA data interpretation.

### Epidemiological Relationships between Bmel3 Isolates in this Study

Although the Alpine ibex is known to be susceptible to *Brucella* infections, the prevalence of brucellosis has been reported as extraordinarily low in this wild species [11], [31]–[32]. For instance, a Bmel3 outbreak, initially misidentified as *B. melitensis* bv 2 (B. Garin-Bastuji, unpublished results) [10], was reported in the 1990s in Gran Paradiso National Park, Italy, with a seroprevalence reaching 4.5% [33]. Surprisingly, the wildlife 2012 surveillance campaign has revealed a very high prevalence within the Alpine ibex population (>45%) of the Bargy Massif [4], despite the absence of any new outbreak in domestic animals and while wild ungulates had been considered, up to now, as an epidemiological dead-end host in Europe.

No host specificity or other associations of a MLVA pattern with a particular feature (clinical samples, year, etc.) were noticed, except for a geographical clustering in some cases. These results are consistent with the historical circulation of various types of strains in the region over time, rather than a predominant clone.

The genotypes harbored by chamois strains from previous outbreaks in other Alpine areas (Dpt. 73 and 05) do not seem to be related to neither the recent domestic and human outbreak, nor to the 2012 wildlife strains, suggesting that various types of strains might have historically circulated within the Alpine chamois populations. As expected, transmission dynamics seems to be in agreement with the breeding practices of domestic ruminants (summer transhumance, boarding-house) within each department. Subsequently, our results reveal the possible existing links between wildlife and domestic animals in the past chamois outbreaks, suggesting spillover from domestic to wild ruminants. Furthermore, this study confirmed that brucellosis cases in wildlife remained local, as previously suggested [5], [8]–[9].

On the basis of genotyping results, consolidated by epidemiological investigations, isolates from the bovine and human 2012 outbreak harbored a unique genotype, which suggests a recent and direct contamination from cattle to human. Interestingly, these strains clustered with local (Dpt. 73 and 74) and former domestic isolates, in particular with the last bovine case in 1999, reported in the same massif. Our typing results together with the absence of domestic infection in this region since 1999 suggest that an imported source can be ruled out. They also suggest that a discrete circulation of Bmel3 within a local secondary reservoir is highly probable.

In agreement with our cut-off interpretation, the isolates from Alpine ibex and chamois in 2012 were closely related to the bovine and human 2012 outbreak, suggesting a potential role of wildlife in the contamination of domestic cattle.

Investigation of a possible role of wild ruminants in domestic brucellosis implies that a spillover from domestic animals and persistence of the infection in wild species could be distinguished [8]. This study allows to conclude on the persistence and circulation of a Bmel3 local clone in the area, over a two decade-period, in the protected population of Alpine ibex. In this species, infection could have been transmitted through closely related clones (sub-cluster C1) from either domestic or even wild animals (*e.g*. chamois HS67). Taking into account the limits of the MLVA interpretation, we might wonder whether the Bmel3 infection in ibex could be due to a more recent contamination by a closely related clone of wild origin (*e.g.* chamois HS04) coming from Italy. Indeed, it is known that the Alpine ibex is able to move on very long distances. The existence of a former outbreak in Italy (Gran Paradiso National Park), although having apparently been eradicated [33], raises the question on a possible transmission from Italy to French wild population. MLVA genotypes of the Italian ibex strain (HS12) and strains of the recent French outbreak belong to the same cluster (respectively, sub-clusters C2 and C1). Nevertheless, the ibex herd of the Bargy Massif shows a highly anthropogenic “sedentary lifestyle”, and no animal movements from or to neighboring mountains have been identified (J. Hars, personal results). Furthermore, ibex populations are closely monitored for *Brucella* infection in neighboring massifs, in France, Italy and Switzerland and no recent brucellosis cases have been reported (JM Le Horgne, personal communications). Thus, the hypothesis of a transmission from the Italian or Swiss wildlife to French ibex appears unlikely.

Interestingly, recent studies pointed the absence of brucellosis outbreaks in wildlife, including regions with moderate or high brucellosis prevalence in domestic animals, except for *B. suis* and *B. abortus* outbreaks [9]. The high brucellosis prevalence reported in wildlife, and especially in ungulates, reflects the direct effects of artificial management on wildlife and brucellosis transmission [34]. High prevalence of *B. suis* bv 2 infection in wild boar and hare has been related to high population densities due to artificial game management [9]. Moreover, prior to strict regulatory programs in Greater Yellowstone (USA), the seroprevalence in bison herd could reach approximately 50% [35]. Recurrent transmission from elk and bison reservoirs to domestic cattle in Greater Yellowstone area highlights the direct consequences of human activities that increase wildlife-livestock interface areas [12].

This situation is similar to that of the protected ibex population in France. The important anthropogenic effects (habitat modifications, increased wildlife density, sedentary behavior, interface areas with domestic animals, salt/mineral feeding that facilitates contacts, etc…) could account for the high brucellosis prevalence found in ibex in the Bargy Massif. Moreover, the genetic integrity of Alpine ibex in the Alpine massifs, strongly modified by anthropic effects, might have also played a role. Indeed, following the almost extinction of ibex in the late 19th century (survival of one single population in the Gran Paradiso Massif, Italy), reintroductions took place into mountain areas in Austria, France, Germany, Italy, Slovenia and Switzerland [36] and reintroduction in the Bargy Massif in the 1970s included only about 15 animals of the same Swiss herd. Since that date, the population of the Bargy Massif has increased to 500 without any foreign blood. All these factors may have contributed to make the Bargy Massif ibex population a semi-domestic population able to self-sustain the B. melitensis infection, with high prevalence, facilitated by the human interventions, which has never been reported before in wild ungulates.

An investigation, that consisted in enclosing during 40 days a 7-year-old male Alpine ibex with brucellosis clinical signs (carpal arthritis and orchitis) with domestic ruminants (sheep and goats) concluded that the transmission of infection from ibex to domestic ruminants by direct and indirect contacts was not very probable [33]. Nevertheless, the major limitation of these experimental conditions concerned the characteristics of the infected animal which only presented closed chronic lesions (testicles, carpal bursa), without any proof of excretion in the environment, restraining the risk of transmission to livestock or among wild ungulates. During the 2012 wildlife investigation, two Bmel3 strains were isolated from urine from two male ibex. Furthermore, a strain was isolated from a vaginal swab of a female ibex, for which no clinical sign had been detected. Therefore, the circulation of *Brucella* among the wild population could have been facilitated by the excretion of bacteria directly in the environment as well as through mating.

Interestingly, on the basis of the 3 U-difference interpretation cut-off, the MLVA-panel2B assay not only segregated serial *Brucella* isolates from distinct clinical samples, but also from the same 13-year-old Alpine ibex ORD84, with severe arthritis clinical signs, into distinct clusters, corresponding to two epidemiological lineages. The strain isolated from urine was closely related to the recent bovine outbreak (only 1 U-difference), while the strain isolated from a calcified arthritis lesion was closely related to the antique domestic outbreaks. Taking account the limits of MLVA interpretation, these data could suggest a Bmel3 co-infection in the same animal.

Despite the high brucellosis prevalence in ibex and the fact that this species share the same pastures and can hybridize with domestic goats [37], no cases in sheep and goats have been observed. As reported for other ibex pathogens [38], the pathogenicity of the *B. melitensis* strain currently circulating among wildlife could be correlated with a host-specificity, *i.e*. (*i*) a better adaptation to the ibex host or (*ii*) an opportunist nature due for instance to co-morbidity, leading to asymptomatic carriage of *B. melitensis* in ibex that could have remained hidden for a long time.

The status of protected species for the Alpine ibex population hinders the potential control and eradication measures, challenging for a better knowledge of local Alpine ibex aspects such as their behavior and possible cohabitation with livestock, animal movements patterns, and population density.

## Conclusion

The MLVA-panel2B strategy provides efficient and sufficient genotyping scheme to track the infection sources and investigate the infection circulation over time in a fine scale setting. As regards the limits of the MLVA interpretation, the genetic diversity of Bmel3 strains, isolated from the French Alps and the neighboring departments/countries, suggests that the recent bovine outbreak and human case could originate from the Alpine ibex population. The anthropogenic factors could account for the high brucellosis prevalence found in ibex in the infected massif. This study describes for the first time a *B. melitensis* spillover from wildlife to domestic ruminants and the infection sustainability in this species, considered as semi-domestic in Europe.

Despite the fact that no information allows to date the spread of the disease into and within the wild reservoir, the high brucellosis prevalence observed within this ibex population reveals that control and surveillance measures applied these last decades were insufficient to identify the disease in wildlife.

## Supporting Information

Table S1 *Brucella melitensis* biovar 3 strains isolated from different sources and of different geographical origins, investigated in this study.
(DOC)
