# Supplementary material for: Brucella melitensis in France: Persistence in Wildlife and Probable Spillover from Alpine Ibex to Domestic Animals
Source: PLoS One. 2014 Apr 14;9(4):e94168. doi: 10.1371/journal.pone.0094168 (PMC3986073; doi:10.1371/journal.pone.0094168)
Supplement: Table S1 — Brucella melitensis biovar 3 strains isolated from different sources and of different geographical origins, investigated in this study. (DOC) [file pone.0094168.s001.doc]

**Table S1**: *Brucella melitensis* biovar 3 strains isolated from different sources and of different geographical origins, investigated in this study.

| **Key** | **Id Strain*** | **Year** | **Host** | **Country** | **Dpt.** | **Sample** | **Details/ Clinical signs/ Risk factors** |
| --- | --- | --- | --- | --- | --- | --- | --- |
| HS01 | 12-68 | 2012 | Human | France | 74 | Blood culture | Domestic and human outbreak 2012. M, 10 years old, without risk factors.  Bargy Massif (Grand Bornand). |
| HS02 | 12-1953 | 2012 | Bovine | France | 74 | Lymph node | Domestic and human outbreak 2012. Dairy cow, 6 years old. Seronegative, Culture positive. Bargy Massif (Grand Bornand). |
| HS03 | 12-1809 | 2012 | Bovine | France | 74 | Lymph node | Domestic and human outbreak 2012. Dairy cow, 10 years old, mother born in 1999. Abortion (due to *Brucella*?). Seropositive. Bargy Massif (Grand Bornand). |
| HS04 | 12-3648 | 2012 | Chamois | France | 74 | Arthritis purulent lesion | Wildlife Surveillance Campaign 2012. F, 7 years old, animal n°ISF7464. Arthritis, important abscess on the foreleg joints. Bargy Massif. |
| HS05 | 12-3777 | 2012 | Alpine ibex | France | 74 | Arthritis purulent lesion | Wildlife Surveillance Campaign 2012. M, n° ORD 083, 11 years old. Arthritis. Bargy Massif (Petit Bornand). |
| HS06 | 12-3973 |  |  |  |  | Urine |  |
| HS07 | 12-3974u | 2012 | Alpine ibex | France | 74 | Urine | Wildlife Surveillance Campaign 2012. M, n°ORD84. 13 years old. Arthritis, foreleg joint, caseous calcification.Bargy Massif (Petit Bornand). |
| HS08 | 12-3974p |  |  |  |  | Arthritis purulent lesion |  |
| HS09 | 12-4041-8027 | 2012 | Alpine ibex | France | 74 | Arthritis purulent lesion | Wildlife Surveillance Campaign 2012. F, 9 years old. Animal n°2012-013. Arthritis, foreleg joint.Bargy Massif (Grand Bornand). |
| HS10 | 12-4150-8305 | 2012 | Alpine ibex | France | 74 | Vaginal swab | Wildlife Surveillance Campaign 2012. F, 8 years old. Animal n°2012-026Bargy Massif. |
| HS11 | 07-415 | 2007 | Human | France? Italy? | 38? | ND** | F, 70 years old. Spondylitis. Frequent trips in Italy. Consumption of raw milk cheese in Isère (Dpt 38) |
| HS12 | 05-1406 | 2005 | Alpine ibex | Italy | ND | ND | Gran Paradiso National Park outbreak (Italy). |
| HS13 | 05-3708 | 2002 | Human | Switzerland | ND | ND | ND |
| HS14 | 04-1362 | 2004 | Human | France? Spain? | 69? | ND | F, 55 years old. Arthritis Veterinary Assistant, Brucellosis 13 years ago (knee prosthesis). New infection or reactivation? Possible origin: Spain. |
| HS15 | 01-617 | 2001 | Ovine | France | 04 | ND | ND |
| HS16 | 01-3016 | 2001 | Bovine | France | 26 | ND | ND |
| HS17 | 01-7279 | 2001 | Chamois | France | 73 | ND | Beaufortain outbreak 1996-2001. Animal n°3782. |
| HS18 | 01-8444 | 2001 | Chamois | France | 73 | ND | Beaufortain outbreak 1996-2001. Animal n°7185. |
| HS19 | 00-2956 | 2000 | Bovine | France | 26 | ND | ND |
| HS20 | 00-3402 | 2000 | Ovine | France | 04 | ND | ND |
| HS21 | 00-3886 | 2000 | Human | France? Italy? | 38? | ND | F, 67 years old, Possible origin: Italy. |
| HS22 | 00-4216 | 2000 | Human | France | 26 | ND | M, 9 years old. Son of the stockbreeder whose herd was recently culled due to *B. melitensis* bv 3 infection. |
| HS23 | 00-8281 | 2000 | Ovine | France | 04 | ND | ND |
| HS24 | 00-8770 | 2000 | Chamois | France | 73 | ND | Beaufortain outbreak 1996-2001. |
| HS25 | 99-5236 | 1999 | Bovine | France | 74 | ND | ND |
| HS26 | 99-6103 | 1999 | Human | France? Italy? | 38? | ND | F, 21 years old, Possible origin: Italy. |
| HS27 | 98-5983 | 1998 | Bovine | France | 38 | ND | Abortion. 8 days before calving date. Holding infected by brucellosis. |
| HS28 | 97-702-215 | 1997 | Bovine | France | 38 | ND | Abortion. Alpine pasture with sheep during summer. |
| HS29 | 97-5848 | 1997 | Human | France? Spain? | 38? | ND | M, 42 years old, abattoir employee. Undulating fever, night sweats. Orchitis. Possible origin: Spain.. |
| HS30 | 97-6816 | 1997 | Chamois | France | 73 | ND | Beaufortain outbreak 1996-2001. M, 4 years old. |
| HS31 | 97-7686 | 1997 | Human | France | 38? | ND | Consumption of goat and cow cheese. |
| HS32 | 96-5489 | 1996 | Human | France | 38 | ND | ND |
| HS33 | 96-5620 | 1996 | Chamois | France | 73 | ND | Beaufortain outbreak 1996-2001. |
| HS34 | 96-10469 | 1996 | Chamois | France | 73 | ND | Beaufortain outbreak 1996-2001. 6.5 years old. |
| HS35 | 96-10470 | 1996 | Chamois | France | 73 | ND | Beaufortain outbreak 1996-2001. |
| HS36 | 96-11593 | 1996 | Chamois | France | 73 | ND | Beaufortain outbreak 1996-2001. |
| HS37 | 95-1185 | 1995 | Bovine | France | 73 | ND | ND |
| HS38 | 95-12127 | 1995 | Bovine | France | 73 | ND | ND |
| HS39 | 94-1243 | 1994 | Ovine | France | 04 | ND | ND |
| HS40 | 94-1599 | 1994 | Ovine | France | 04 | ND | ND |
| HS41 | 94-2240 | 1994 | Ovine | France | 04 | ND | ND |
| HS42 | 94-3153 | 1994 | Bovine | France | 05 | ND | ND |
| HS43 | 94-8798 | 1994 | Bovine | France | 05 | ND | ND |
| HS44 | 94-10675 | 1994 | Bovine | France | 05 | ND | ND |
| HS45 | 94-10677 | 1994 | Bovine | France | 05 | ND | ND |
| HS46 | 93-219 | 1993 | Bovine | France | 05 | ND | ND |
| HS47 | 93-401 | 1993 | Caprine | France | 73 | ND | ND |
| HS48 | 93-2793 | 1993 | Chamois | France | 73 | ND | Lautaret outbreak 1982-1993 |
| HS49 | 92-101 | 1992 | Bovine | France | 05 | ND | ND |
| HS50 | 92-1142 | 1992 | Bovine | France | 26 | ND | ND |
| HS51 | 92-1902 | 1992 | Bovine | France | 73 | ND | ND |
| HS52 | 92-1903 | 1992 | Bovine | France | 73 | ND | ND |
| HS53 | 92-2560 | 1992 | Ovine | France | 04 | ND | Abortion |
| HS54 | 92-2805 | 1992 | Caprine | France | 04 | ND | ND |
| HS55 | 92-3557 | 1992 | Bovine | France | 74 | Milk | Dairy cow |
| HS56 | 92-3911 | 1992 | Bovine | France | 38? | ND | Abortion |
| HS57 | 92-4207-1 | 1992 | Bovine | France | 05 | ND | ND |
| HS58 | 92-6310-1 | 1992 | Bovine | France | 73 | ND | ND |
| HS59 | 92-12433 | 1992 | Bovine | France | 73 | ND | ND |
| HS60 | 91-1848 | 1991 | Bovine | France | 73 | ND | ND |
| HS61 | 91-6713 | 1991 | Bovine | France | 05 | ND | ND |
| HS62 | 91-9258 | 1991 | Bovine | France | 73 | ND | ND |
| HS63 | 91-10647-1207 | 1991 | Bovine | France | 38 | ND | ND |
| HS64 | 91-14129-10594 | 1991 | Bovine | France | 74 | ND | ND |
| HS65 | 91-14417 | 1991 | Bovine | France | 73 | ND | ND |
| HS66 | 90-13312 | 1990 | Chamois | France | 05 | ND | Lautaret outbreak 1982-1993 |
| HS67 | 01-9337 | 2001 | Chamois | France | 73 | ND | Beaufortain outbreak 1996-2001. Testicle. Animal n°128 |
| HS68 | 95-2748 | 1995 | Ovine | France | 06 | ND | ND |
| HS69 | 91-15250-406 | 1991 | Bovine | France | 73 | ND | ND |
| HS70 | 91-12140-3106-3 | 1991 | Bovine | France | 05 | ND | ND |
| HS71 | 91-7100 femelle | 1991 | Canine | France | 05 | ND | ND |
| HS72 | 91-3390 | 1991 | Caprine | France | 06 | ND | ND |
| HS73 | 90-10858-œil | 1990 | Chamois | France | 05 | ND | Lautaret outbreak 1982-1993 |
| HS74 | 90-3934 | 1990 | Ovine | France | 04 | ND | ND |
| HS75 | 89-7452 | 1989 | Canine | France | 38 | ND | ND |
| HS76 | 89-2456 | 1989 | Ovine | France | 73 | ND | ND |
| HS77 | 89-2859 | 1989 | Ovine | France | 04 | ND | ND |
| HS78 | 16M |  |  |  |  | ND | *B. melitensis* biovar 1 reference strain |
| HS79 | Ether | 1961 | Caprine | Italy |  | ND | *B. melitensis* biovar 3 reference strain |

* IdStrain: Strain Identification; M: male; F: female; Dpt.: department.

** ND: no data available.
